# Retinoic Acid Downregulates HSPB8 Gene Expression in Human Breast Cancer Cells MCF-7

**DOI:** 10.3389/fonc.2021.652085

**Published:** 2021-05-31

**Authors:** Margherita Piccolella, Riccardo Cristofani, Barbara Tedesco, Marta Chierichetti, Veronica Ferrari, Elena Casarotto, Marta Cozzi, Valeria Crippa, Paola Rusmini, Mariarita Galbiati, Angelo Poletti, Elio Messi

**Affiliations:** ^1^ Dipartimento di Scienze Farmacologiche e Biomolecolari (DiSFeB), Centro di Eccellenza sulle Malattie Neurodegenerative, Università degli Studi di Milano, Milan, Italy; ^2^ Unit of Medical Genetics and Neurogenetics, Fondazione IRCCS Istituto Neurologico Carlo Besta, Milan, Italy

**Keywords:** retinoic acid, HSPB8, breast cancer, miRNAs, proliferation

## Abstract

Breast cancer (BC) is a serious and widespread disease for which different treatments have been developed. In addition to the classic therapies, the treatment with retinoic acid (RA) is still being clinically investigated. RA reduces cancer cells proliferation and migration, but its molecular mechanism of action is not clear. In tumor development, autophagy promotes cancer cell survival and prevents apoptosis. Small heat shock protein B8 (HSPB8) acts together with its co-chaperone BCL-2 associated athanogene 3 (BAG3) stimulating BC proliferation and migration. We analyzed whether direct correlations exist between RA and HSPB8 or BAG3 and how this may play a role in BC. We measured HSPB8 and BAG3 gene expression in MCF-7 BC cells and we analyzed the potential correlation between the antiproliferative and antimigratory effect of RA with the expression level of HSPB8. We found that in MCF-7 cells RA reduces both HSPB8 and BAG3 gene expression and it alters the mitotic spindle organization. Notably, the effects of RA on HSPB8 levels are exerted at both transcriptional and translational levels. RA effects are possibly mediated by miR-574-5p that targets the HSPB8 transcript. Our results suggest that therapeutic doses of RA can efficiently counteract the adverse effects of HSPB8 in BC progression.

## Introduction

Breast cancer (BC) is both the most frequent cancer and the main cause of cancer-related death among women (1). A majority (~70%) of breast tumors express estrogen receptor (ER), and a significant fraction (~90%) of these ER-positive (ER^+^) breast tumors is also androgen receptor-positive (AR^+^) (2). It is now clear that ER^+^ BC patients should undergo specific anti-hormonal therapy (3) but, unfortunately, ~30-50% of initially responsive patients develop resistance to therapies (2), leading to the appearance of more aggressive tumor forms (2, 4).

Retinoids are vitamin A metabolites which act as differentiating agents, cell cycle progression inhibitors, and apoptosis inducers leading to growth arrest in different human cancer cell lines (5). A specific retinoid, all-*trans* retinoic acid (RA) is a standard therapy for promyelocytic leukemia, but its use in solid cancer is still controversial (6). Unfortunately, few clinical investigations related to RA anticancer activity are available (7). Retinoids act through two subfamilies of nuclear retinoid receptors (RARs and RXRs) that belong to the family of steroid/thyroid hormone receptors. To date, six different genes encoding for nuclear RAR/RXRs have been identified. RAR/RXRs are ligand-activated transcription factors (TF) able to bind RA responsive elements (RAREs) located in the promoter of responsive genes (7). The interplay between RAR and ER has been reported. Indeed, both receptors can bind the same DNA responsive elements and in ER^+^-MCF-7 cells they act antagonistically to regulate human BC-associated genes (8). Interestingly, also microRNAs (miRNAs) like miR-210, miR-23a/24-2, miR-17/92, miR-424/450b are antagonistically regulated by both estrogen and RA in MCF-7 cells (9). Noteworthily, RA treatment reduces the proliferation of ER^+^-MCF-7 but not of MDA-MB-231 cells, which are ER^-^; at the same time, RA upregulates the expression of the pro-oncogenic miR-21 which reduces cellular motility, despite its ability to counteract RA antiproliferative activity (10). A study carried out on the ER^-^ SKBR3 cells showed that RA treatment modulates the expression of a considerably high number of miRNAs (11). Chemoresistance of BC cells has been recently correlated with autophagy impairment mediated by miR-27a expression (12). Autophagy is one of the intracellular degradative systems responsible for the clearance of damaged proteins, and organelles (13), and it is apparently involved in the generation of drug-resistant BC cells (14). In cancer cells, autophagy initially has a suppressive activity, but subsequently it can increase tumor cell survival by enhancing stress tolerability, perturbing cell function and reducing apoptotic cell death. Chaperone-assisted selective autophagy (CASA) is a peculiar form of autophagy that exerts protective mechanisms against human diseases (15). A crucial player in CASA is the small Heat Shock Protein B8 (HSPB8), which acts as autophagy flux enhancer (16), together with its co-chaperone BCL2-associated athanogene 3 (BAG3), the Heat Shock Cognate 70 Protein (HSC70) and the E3-ubiquitin protein ligase CHIP (15, 17, 18). This CASA complex recognizes aberrant proteins and drives them to autophagosomes for their clearance (17–19). HSPB8 is a limiting component of the CASA complex (20), and its expression is associated with increased proliferation and migration of MCF-7 cells (21). Thus, HSPB8 plays a relevant role in the modulation of MCF-7 cell aggressiveness, and this action correlates with estrogen activity (21). While it is well-known that the retinoid system acts as a biological antagonist of the estrogenic system, among all the data collected about the effects of RA in BC none has been focused on its possible modulation of HSPB8 expression (16). The aim of this work was to analyze the direct effect of RA on HSPB8 expression in MCF-7 cells and its possible role in preventing the adverse effects of HSPB8 in BC. We found that RA reduces *HSPB8* transcription and translation by acting on its promoter and on its mRNA stability, and this results in a disruption of the correct structure of the mitotic spindle. Our data also showed that the *HSPB8* mRNA could be targeted in its open reading frame (ORF) by miRNAs that can regulate *HSPB8* mRNA stability, one of which is miR-574-5P. Thus, RA may be viewed as a potent physiological antagonist of HSPB8 adverse activities in BC.

## Materials and Methods

### Chemicals

17β-estradiol (#E1024), all-*trans* retinoic acid (RA; #R2625) and ICI 182.780 (#I4409) were obtained from Sigma-Aldrich (St. Louis, MO, USA).

### Cell Culture and Treatments

MCF-7 and MDA-MB-213 cells were originally obtained from the American Type Culture Collection (Rockville, MD) and are routinely used in our laboratory between passages 8 and 12 (21) (5% CO_2_, 37° C, humidity > 90%). Short-tandem repeat (STR) profile has been performed by Eurofins Genomics Europe (Ebersberg, Germany). Genetic characteristics were determined by PCR-single-locus-technology. 16 independent PCR-systems D8S1179, D21S11, D7S820, CSF1PO, D3S1358, TH01, D13S317, D16S539, D2S1338, AMEL, D5S818, FGA, D19S433, vWA, TPOX, and D18S51 were investigated (**Table 1**). Before any experimental procedure, medium was replaced overnight with RPMI 1640 without fetal bovine serum (FBS) and without phenol red to synchronize cell growth. In all experiments, 17β-estradiol was used at the dose of 10nM and RA at doses ranging from 0.01μM to 1μM accordingly to literature (21–23). Plasmid transfection was performed on the third day of RA treatment. The immortalized motoneuronal NSC-34 cells were obtained from Niel Cashman and are routinely used in our laboratory between passages 10 and 20 (24, 25).

**Table 1:** Cell Line Authentication. The table shows the result of the cell line analysis.

Cell Line Authentication Report		
Sample Name	MCF-7	MDA-MB-231
D8S1179	10,14	13,13
D21S11	30,30	30,33.2
D7S820	8,9	8,8
CSF1PO	10,10	12,13
D3S1358	16,16	16,16
TH01	6,6	7,9.3
D13S317	11,11	13,13
D16S539	11,12	12,12
D2S1338	21,23	20,21
D19S433	13,14	11,14
vWA	14,15	15,15
TPOX	9,12	8,9
D18S51	14,14	11,16
AMEL	X,X	X,X
D5S818	11,12	12,13
FGA	23,25	22,23
Database Name	MCF-7	MDA-MB-231

### Plasmids, miR-Inhibitor and Transfection

pCMV-β-gal plasmid was obtained from Clontech Lab (Mountain View, CA, USA). hPromB8-LUC plasmid contains the firefly luciferase cDNA under the control of a -3000/+523 human *HSPB8* promoter region (18); pCI-hHSPB8-wild-type (wt) codes for the human HSPB8 protein (26). pHSPB8-mut has been obtained in our laboratory by exchanging the *ApaI/SalI* coding fragment with the mutated sequence obtained from Eurofins Genomics. pEGFP-G93A-SOD1 expresses the green fluorescent protein (GFP)-tagged mutant G93A SOD1 (17). pcDNA3.1 (Life Technologies, #V790-20) plasmid was used to normalize the amount of transfected plasmid DNA. All plasmids were transfected in MCF-7 and MDA-MB-213 cell lines as previously described (21). NSC-34 cells were transfected as previously described (25). The hsa-miR-574-5p miRCURY LNA miRNA Inhibitor (Qiagen) was used to inhibit miR-574-5p activity, and the miRCURY LNA miRNA Inhibitor Control (Qiagen) was used as a control. Both miRNAs were transfected at the final concentration of 50nM according to the manufacturer's instructions.

### RT-qPCR Analysis

MCF-7 and MDA-MB-231 cells were seeded in 6-well plates at 300,000 cells/well and treated for 2 or 3 days with increasing doses of RA (0.01-1μM) and with 17β-estradiol (10nM). Then, cells were harvested in 300 µL TRI Reagent (Sigma-Aldrich; #T9424) and total RNA was isolated. 1μg total RNA was treated with DNAse and reverse-transcribed into cDNA using the High-Capacity cDNA Archive Kit (Applied Biosystems, Life Technologies Corporation; #4368813). Primers were synthetized by Eurofins Genomics with the sequences reported in **Table 2**. Real-time PCR (qPCR) was performed as previously described (21). Data were transformed using the equation 2^−ΔΔCt^ to give N-fold changes in gene expression; all statistical analyses were performed with ΔCt values. Each sample was analyzed in triplicate (n=3); *HSPB8* and *BAG3* values were normalized to those of Ribosomal Protein Lateral Stalk Subunit P0 (*RPLP0*).

**Table 2:** Primer List.

Gene		Sequence (5’-3’)
HSPB8	forward	AGAGGAGTTGATGGTGAAGACC
	reverse	CTGCAGGAAGCTGGATTTTC
BAG3	forward	GGGTGGAGGCAAAACACTAA
	reverse	AGACAGTGCACAACCACAGC
RPLP0	forward	GTGGGAGCAGACAATGTGGG
	reverse	TGCGCATCATGGTGTTCTTG

### Western Blot Assay

MCF-7 and MDA-MB-231 cells were seeded in 6-well plates at 300,000 cells/well and treated for 2 or 3 days with increasing doses of RA (0.01-1μM) and with 17β-estradiol (10nM). Western blot (WB) assay was performed as previously described (21). NSC-34 cells were seeded in 12-well plates at 80,000 cells/well. 48h after transfection, cells were harvested and centrifuged for 5 min at 100 × *g* at 4°C; the cell pellets were then re-suspended in Phosphate-Buffered Saline (PBS) (Sigma-Aldrich) added with a protease inhibitor cocktail (Sigma-Aldrich, P4417) and homogenized using slight sonication to lyse cells and nuclei. Total protein concentration was determined using the bicinchoninic acid method (BCA assay; Euroclone, #EMP014500). Equal amounts of proteins (15-20µg) were resolved by electrophoresis on a 10-15% SDS-polyacrylamide gel (SDS-PAGE). Proteins were transferred to 0.45 μm nitrocellulose membranes using a transfer apparatus (Mini Trans-Blot Cell; Bio-Rad Laboratories). The membranes were then processed as previously described (21). For HSPB8, Glyceraldeyde 3-Phosphate Dehydrogenase (GAPDH) and α−Tubulin detection, overnight incubation at 4°C was performed respectively with antibodies listed in **Table 3**. Membranes were then washed and incubated for 1 h at room temperature with secondary antibodies conjugated to horseradish peroxidase (**Table 3**). Immunoreactive bands were visualized using enhanced chemiluminescence detection kit reagents (Westar Antares; Cyanagen, #XLS142). A ChemiDoc XRS System (Bio-Rad) was used for image acquisition.

**Table 3:** Antibodies list.

Antibody	Species	Dilution	Application	Company (Catalog #)
HSPB8	rabbit	1:2,000	WB	kindly provided by Jacques
		1:200	IF	Landry, Quebec, Canada
BAG3	rabbit	1:10,000	WB	Abcam; #ab47124
		1:1,000	IF	
α-Tubulin	mouse	1:4,000	WB	Sigma-Aldrich; #T6199
		1:200	IF	
GFP	mouse	1:1000	WB/FRA	Immunological Sciences; #MAB94345
Anti-rabbit	goat	1:10,000	WB (HSPB8)	Santa Cruz Biotech; #E2908
		1:20,000	WB (BAG3)	
Anti-mouse	goat	1:10,000	WB (α-Tubulin)	Santa Cruz Biotech; #H2704
Anti-rabbit	donkey	1:500	IF (red)	Rockland; #611-700-127
Anti-mouse	donkey	1:500	IF (green)	Rockland; #710-702-124
Anti-rabbit	goat	1:200	IF (green)	Thermo Fisher; #A11070

### Immunofluorescence Analysis

MCF-7 cells were plated on 13mm-diameter coverslips at 50,000 cells/well and treated with RA (1μM) for 2 or 3 days, then fixed in 4% paraformaldehyde, permeabilized in 0.5% Triton X-100 and treated with 5% FBS (GIBCO) in PBS. Subsequently, cells were incubated overnight at 4°C with the primary antibodies listed in **Table 3**. Incubation with secondary antibodies (**Table 3**) was performed for 1 h at room temperature. Nuclei were stained with Hoechst 33342 (Thermo Fisher). Finally, the coverslips were mounted with Mowiol 40-88 (Sigma-Aldrich). Images were collected by UIC-Metavue 6.2.2 (UIC-Crisel Instr. Rome) imaging system on an Axiovert Zeiss 200 microscope, utilising a 40× magnification (NA 0.8) objective.

### Cell Growth Studies

To study the effect of RA and 17β-estradiol on MCF-7, cells were seeded in 24-well plates at 40,000 cells/well and treated up to 6 days with RA (1μM) and 17β-estradiol (10nM); cell growth/viability was measured by MTT [3-(4,5-dimethylthiazol-2-yl)-2,5-diphenyltetrazolium bromide] assay. Briefly, culture medium was replaced with fresh medium containing MTT (1.5 mg/ml) and the multiwells were incubated at 37°C for 1 h, then the medium was removed and 2-propanol (500μl) was added to solubilize the crystals. The absorbance was read at 550 nm with an Enspire 2300 Multimode Plate Reader (Perkin Elmer, Italy) (21).

### miRNA RT-qPCR Analysis

10ng of total RNA were reverse-transcribed and amplified using the miRCURY LNA miRNA PCR Starter Kit (Qiagen, Ref 339320. The kit includes a spike-in control primer set (UniSp6), UniSP6 RNA Spike-in-template, one candidate endogenous control primer set (miR-103a-3p) and two validated primer sets, which in our case were miR-297 (Qiagen YP00206079) and miR-574-5p (Qiagen YP02116206). As additional control micro-RNAs (miRNAs), we chose miR-25-3p (Qiagen YP00204361) and miR-331-3p (Qiagen YP00206046) because both are used as markers in BC analysis (27, 28) and their expression is not modified by RA treatment in the BC cell line SKBR3 (11). Real-time PCR was performed with miRCURY LNA SYBR Green Master Mix (Qiagen) in 10μL total volume using the CFX 96 Real Time System (Bio-Rad). The expression of target miRNAs miR-297 and miR-574-5p was normalized against miR-25-3p, miR-103a-3p, miR-331-3p and UniSp6 using the 2^-ΔΔCt^ method. To validate the real-time system used for miRNA analysis, we measured the levels of UniSp6RNA, a control RNA provided with the Starter Kit, that was added before the reverse transcription in equal amount to all samples (see Supplementary material).

### Transcriptional Activity

Transcriptional activity was measured using the LucLite Kit from Perkin Elmer (Waltham, MA, USA). MCF-7 cells treated with RA (1μM) and 17β-estradiol (10nM) were plated in 24-well plates at a density of 100,000 cells/well and transfected with 0.4μg pCMV-β-gal plasmid and with 0.6μg hPromB8-LUC plasmid. Each sample was analyzed in sextuplicate. All plasmids were transfected as described above. The inducible firefly luciferase activities controlled by the HSPB8 promoter have been normalized using the constitutive β-galactosidase activities produced under the control of the Citomegalovirus (CMV) promoter (by co-transfecting pCMV-β-gal). The luminescence was evaluated using Wallac 1450 MicroBeta TriLux (Perkin Elmer, Waltham, MA, USA). β-galactosidase activity (coded by pCMVβ) was then assayed in the same samples. For β-galactosidase 50μl of each sample were added to 750μl Assay Buffer in presence of 4mg/ml β-galactosidase substrate o-nitrophenyl-β-D-galactopyraniside (ONPG, Sigma) and incubated at 37°C until yellow color appeared. Then, 500μL Na_2_CO_3_ (1M) were added, 200μL of the final solution were transferred to 96-well plates and 420nm absorbance was quantified using EnSpire 2300 Multimode Plate Reader (Perkin Elmer, Italia).

### Filter Retardation Assay

NSC-34 cells were plated at 80,000 cells/well in 12-well plates, transfected and collected 48h after transfection in PBS added with protease inhibitor. Cells were homogenized using slight sonication to lyse cells and nuclei as previously described (29). Filter retardation assay (FRA) was performed using a Bio-Dot SF Microfiltration Apparatus (Bio-Rad). 6μg of the total protein extracts were filtered through 0.22μm cellulose acetate membranes (Whatman, 100404180). The membranes were probed as described for WB. A ChemiDoc XRS System (Bio-Rad) was used for image acquisition. The optical density of samples assayed with WB or FRA was detected and analyzed using the Image Lab software (Bio-Rad). Statistical analyses were performed using relative optical densities, defined as the ratio between the optical density of each independent biological sample (n = 3) and the mean optical density of control samples.

### Migration Assay

Briefly, cell migration assay was performed using a 48 well-Boyden chamber (NeuroProbe, Inc., Gaithersburg, MD, USA) containing 8µm polycarbonate filters (Nucleopore, Concorezzo, Milan, Italy). Filters were coated on one side with 50µg/ml laminin rinsed once with PBS, and then placed in contact with the lower chamber containing RPMI 1640 medium. MCF-7 cells, overexpressing mutated HSPB8 for 3 days and treated with 1μM RA up to 6 days, were collected, added in aliquots (75,000 cells/50µl) to the top of each chamber and allowed to migrate through coated filters for 4h. At the end of the incubation, the migrated cells attached on the lower membrane surfaces were fixed, stained with Diffquik (Biomap, Italy) and counted in standard optical microscopy (21).

### Statistical Analysis

Statistical analysis was performed by one-way ANOVA followed by Bonferroni multiple comparison tests. *p<0.05 was considered statistically significant. Computations were performed with the PRISM (ver. 8.2.1) software (GraphPad Software, LaJolla, CA, USA).

## Results

### Effect of RA on HSPB8 and BAG3 Gene Expression

**Figure 1:**
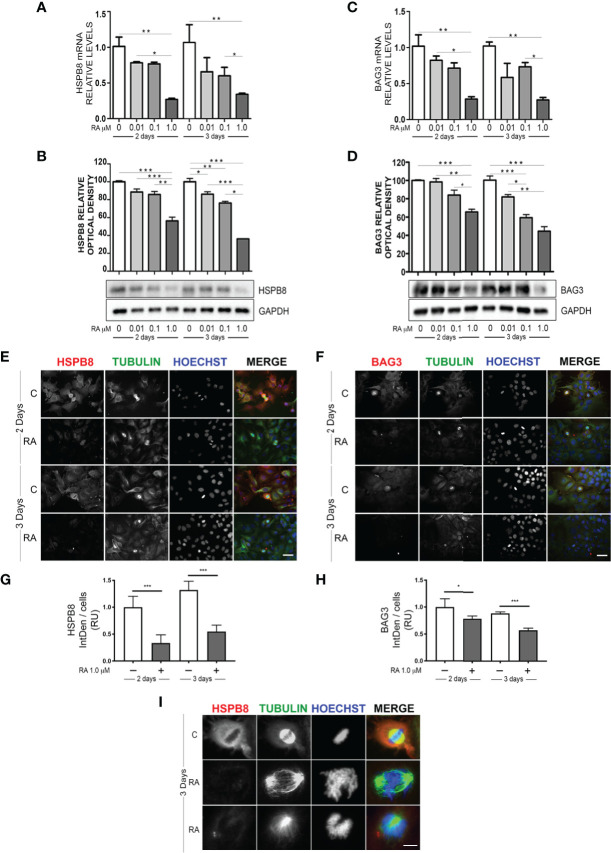
Effect of RA treatment in MCF-7 cells. HSPB8 **(A, B)** and BAG3 **(C, D)** mRNA and protein levels measured by RT-qPCR analysis and western blot analysis in MCF-7 cells treated for 2 and 3 days with different doses of RA. **(E)** Immunofluorescence analysis of HSPB8 (red) and tubulin (green) in MCF-7 cells treated for 2 and 3 days with 1μM RA, nuclei were stained with Hoechst (scale bar = 20μm). **(F)** Immunofluorescence analysis of BAG3 (red) and tubulin (green) in MCF-7 cells treated for 2 and 3 days with 1μM RA (scale bar = 20μm). **(G, H)** Fluorescent intensity quantification of HSPB8 and BAG3, nuclei were stained with Hoechst. **(I)** Higher magnification of the mitotic spindle (scale bar = 5μm). *p < 0.05, **p < 0.01 and ***p < 0.005 in all charts. Graph bars represent the mean of three independent experiments.

We initially evaluated whether RA may modulate HSPB8 expression in MCF-7 cells. For this purpose, we treated MCF-7 with increasing doses of RA for 2 or 3 days, accordingly to the literature (23). The analysis of *HSPB8* gene expression, performed using RT-qPCR on MCF-7 cells, is reported in **Figure 1A**. The data clearly show that both 2 and 3 days of 1μM RA treatment resulted in a significant reduction of *HSPB8* mRNA levels, while lower doses were not able to modify *HSPB8* expression at both times considered. A similar result was observed for HSPB8 protein by western blot (WB) analysis (**Figure 1B**): in fact, no significant modulation of HSPB8 protein levels was observed after 2 and 3 days of treatment with the lowest doses of RA, while 1μM RA treatment reduced HSPB8 levels. Similarly, also BAG3 mRNA and protein levels were reduced by RA (**Figures 1C, D**). Moreover, we performed Immunofluorescence (IF) analysis to evaluate HSPB8, BAG3 and tubulin intracellular localization (**Figures 1E, F**). HSPB8 and BAG3 intracellular distribution in untreated cells was in line with our previous observations (21). Interestingly, in MCF-7 cells treated with 1μM RA the HSPB8 and BAG3 IF reactivities were significantly reduced at both times considered (**Figures 1E–H**), while they were easily detectable in untreated cells. No changes in tubulin levels and distribution were observed after 2 days of RA treatment, but several dividing cells displayed a different organization of their normal mitotic asset after 3 days of RA treatment. As described by Fuchs and colleagues (30), HSPB8 has a very peculiar localization at metaphase, since it surrounds the complex microtubule spindle network and concentrates adjacent to, but not co-localizing with the chromosomal DNA packed at the metaphase plate. In **Figure 1I**, we showed that HSPB8 reduction induced by RA treatment was associated with the alteration of the mitotic spindle, that appeared highly disorganized, impairing the correct chromosomes alignment. Therefore, our data suggest that the effects of HSPB8 on microtubules could be more relevant during the mitotic phase than during interphase, since the alteration on the tubulin network induced by HSPB8 downregulation was present only on the microtubules forming the mitotic spindle.

### Dual Activity of RA on HSPB8 Transcript Levels

**Figure 2:**
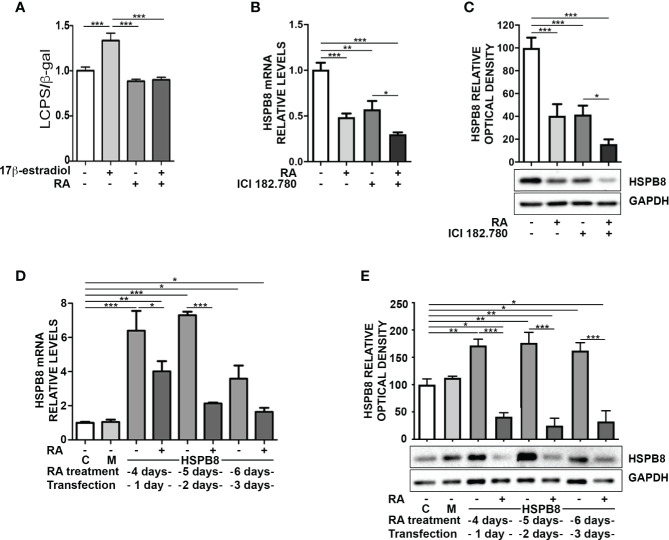
Promoter independent modulation of HSPB8 is induced by RA in MCF-7 cells. **(A)** Transcriptional activity assay of MCF-7 cells treated with 1μM RA and/or 10nM 17β-estradiol transfected with hPromB8-LUC and pCMV-β-gal. Luciferase activity is expressed as luminescence counts per second (LCPS) normalized on β-galactosidase (β-gal) expression. HSPB8 mRNA **(B)** and protein levels **(C)** measured by RT-qPCR analysis and western blot analysis in MCF-7 cells treated for 2 days with 1μM RA and/or 100nM ICI 182.780. **(D)** HSPB8 mRNA levels measured by RT-qPCR analysis in MCF-7 cells. **(E)** HSPB8 protein levels measured by western blot analysis in MCF-7 cells. For D, E untreated cells **(C)**, mock-transfected cells (M) or cells treated up to 6 days with 1μM RA and transfected at day 3 with the plasmid overexpressing wild-type HSPB8. Analyses were performed at 1, 2 and 3 days after transfection. *p < 0.05, **p < 0.01 and ***p < 0.005 in all charts. Graph bars represent the mean of three independent experiments.

The reduction of *HSPB8* gene expression operated by RA suggests that there may be a direct action on the promoter region of the human *HSPB8* gene. To evaluate this hypothesis, we took advantage of the reporter plasmid hPromB8-LUC, in which the cDNA coding for luciferase is placed under the control of the human *HSPB8* promoter (18). We transfected MCF-7 cells with the plasmid and analysed RA effect on luciferase expression, both in basal condition and in the presence of 10nM 17β-estradiol, used as positive control (21) (**Figure 2A**). At 2 days of treatment, we found that RA had no effect in basal condition, whilst it significantly reduced 17β-estradiol-induced luciferase activity. Consistently, we confirmed that, in our experimental condition, RA treatment was able to reduce cell proliferation induced by 17β-estradiol treatment, as already published by Salvatori and colleagues (23) (**Figure S2**).

To investigate the possible correlation between RA and 17β-estradiol in the modulation of *HSPB8* expression, we inhibited ER activation in MCF-7 with the ER antagonist ICI 182.780 and analysed *HSPB8* expression by RT-qPCR (**Figure 2B**) and WB (**Figure 2C**). The data clearly showed that both ICI 182.780 and RA treatments resulted in a significant reduction of *HSPB8* mRNA levels; moreover ICI 182.780 and RA co-treatment showed a synergic effect (**Figure 2B**). A similar result was observed for HSPB8 protein levels (**Figure 2C**), suggesting that ER is necessary to maintain HSPB8 expression, but RA effects might be exerted independently from the ER activity on *HSPB8* promoter (21).

Then, we assessed the possibility that RA effects on HSPB8 expression levels were independent from *HSPB8* promoter regulation. For this purpose, we transfected MCF-7 cells with a plasmid in which *HSPB8* expression is regulated by the Citomegalovirus (CMV) promoter. We found that, also in this case, RA reduced both mRNA (**Figure 2D**) and protein levels (**Figure 2E**) of overexpressed HSPB8 in MCF-7 cells at every time considered. Moreover, we confirmed that this effect was specific for HSPB8, since no modulation was observed on the β-galactosidase enzyme expressed under the control of CMV promoter (**Figure S3**).

Therefore, the RA-mediated reduction of HSPB8 levels might depend on a specific RA-regulated factor, that might act at mRNA level, like for example a micro-RNA (miRNA).

### miRNA Analysis in MCF-7 and MDA-MB-231 Cells and Effects of RA Treatment

**Figure 3:**
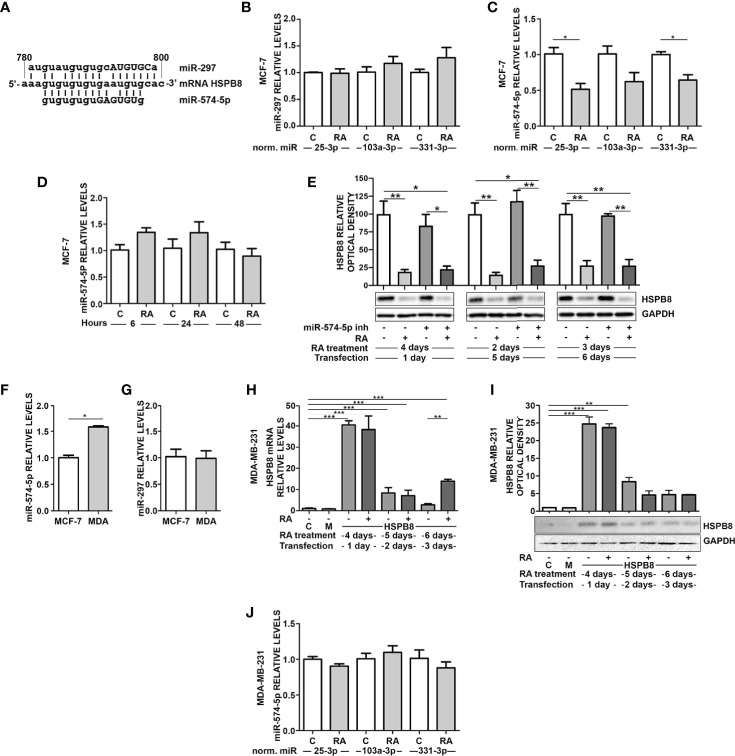
RA modulates miRNAs in MCF-7 and MDA-MB-231 cells. **(A)** Alignment of miR297 and miR-574-5p RNAs with wild-type HSPB8 mRNA ORF region (between 780 and 800 bp), capital letters indicate the *seed sequence* of the two miRNAs. **(B)** RT-qPCR analysis of miR-297 in MCF-7 cells. **(C)** RT-qPCR analysis of miR-574-5p in MCF-7 cells. **(D)** RT-qPCR analysis of miR-574-5p in MCF-7 cells treated for 6h, 24h, and 48 h with 1μM RA, data are normalized utilizing miR-25-3p. **(E)** HSPB8 protein levels measured by western blot analysis in MCF-7 cells treated up to 6 days with 1μM RA and 1, 2 and 3 days after transfection with miR-574-5p-inhibitor. **(F)** RT-qPCR analysis of miR-574-5p in MCF-7 and MDA-MB-231 (MDA) cells, data are normalized utilizing RNA UniSp6 levels. **(G)** RT-qPCR analysis of miR-297 in MCF-7 and MDA-MB-231 (MDA) cells, data are normalized utilizing RNA UniSp6 levels. **(H)** HSPB8 mRNA levels measured by RT-qPCR analysis in MDA-MB-231 cells. **(I)** HSPB8 protein levels measured by western blot analysis in MDA-MB-231 cells. For **(H, I)** untreated cells (C), mock-transfected cells (M) or cells treated up to 6 days with 1μM RA and transfected at day 3 with the plasmid overexpressing wild-type HSPB8. Analyses were performed at 1, 2 and 3 days after transfection. **(J)** MDA-MB-231 cells treated for 4 days with 1μM RA. All miRNAs RT-qPCR analysis are normalized utilizing miR-25-3p, miR-103a-3p or miR-331-3p as housekeeping microRNA. *p < 0.05; **p < 0.01; ***p < 0.005 in all charts. Graph bars represent the mean of three independent experiments.

Our data suggest that endogenous and overexpressed *HSPB8* could be the target of one or more miRNAs, possibly modulated by RA in MCF-7 cells. It is expected that a putative miRNA should be capable of binding in the *HSPB8*-mRNA open reading frame (ORF); in fact, the transcript derived from pCI-hHSPB8 lacks the 5’-untranslated region (5’-UTR) and the 3’UTR of endogenous *HSPB8* mRNA. By scanning *HSPB8* cDNA *in silico* (http://www.mirbase.org) for sequences complementary to known miRNAs, we identified some putative miRNAs that target the ORF region of *HSPB8* mRNA (**Table 4**). Of these, only miR-297 and miR-574-5p were confirmed by a subsequent analysis (http://ophid.utoronto.ca/mirDIP). Both are able to bind *HSPB8* mRNA region comprised between 781bp and 799bp (**Figure 3A**).

**Table 4:** miRNAs targeting HSPB8 ORF.

Accession	ID	Query start	Query end	Subject start	Subject end	Strand
MIMAT0027355	hsa-miR-6727-5p	37	55	1	19	–
MIMAT0027665	hsa-miR-6882-3p	75	93	6	24	+
MIMAT0000722	hsa-miR-370-3p	436	454	2	20	+
**MIMAT0004450**	**hsa-miR-297**	**290**	**308**	**1**	**19**	**+**
MIMAT0023700	hsa-miR-6075	35	53	2	20	–
MIMAT0025474	hsa-miR-6509-5p	22	40	2	20	–
MIMAT0025475	hsa-miR-6509-3p	22	40	1	19	+
MIMAT0027587	hsa-miR-6842-3p	158	176	2	20	+
**MIMAT0004795**	**hsa-miR-574-5p**	**292**	**306**	**8**	**22**	**+**

In bold are highlighted the miRNA confirmed by the analysis done with mirDIP. The table shows the result of *in silico* analysis of HSPB8 ORF with miRbase database.

We thus measured the levels of the two selected miRNAs in MCF-7. miR-297 levels did not show any significant variation upon 1μM RA treatment for 4 days (**Figure 3B**). Otherwise, the same treatment caused a significant reduction of miR-574-5p levels (**Figure 3C**). In order to exclude a possible RA effect on control miRNA levels, we used 3 different control miRNAs (miR-25-3p, miR-331-3p, and miR-103a-3p) to normalize the results. Notably, the reduction of miR-574-5p levels in MCF-7 cells was observed in all the analyzed conditions. Moreover, at 6h, 24h, and 48h of RA treatment we did not observe miR-574-5p reduction (**Figure 3D**).

Then, we treated MCF-7 cells with hsa-miR-574-5p miRCURY LNA miRNA inhibitor, but unfortunately we did not observe a modulation of HSPB8 protein levels after its overexpression (**Figure 3E**). Despite this, we assayed the levels of miR-297 and miR-574-5p also in the ER^-^ MDA-MB-231 cell line that expresses low levels of HSPB8. miRNA levels in the two cell lines have been compared using UniSP6RNA as external reference to normalize samples. Interestingly, miR-574-5p levels were significantly higher in MDA-MB-231 cells compared to MCF-7 cells (**Figure 3F**). No differences were observed in the case of miR-297 levels (**Figure 3G**).

This evidence led us to test the effect of RA on overexpressed HSPB8 also in MDA-MB-231 cells. Data showed that RA treatment did not affect *HSPB8* mRNA (**Figure 3H**) and protein levels (**Figure 3I**). Consistently, we did not observe changes in miR-574-5p in MDA-MB-231 cells after exposure to RA (**Figure 3J**). miR-297 levels did not change in the same experimental conditions (**Figure S4**).

### Effect of RA on MCF-7 Cell Proliferation and Migration in Presence of HSPB8 Variant Overexpression

**Figure 4:**
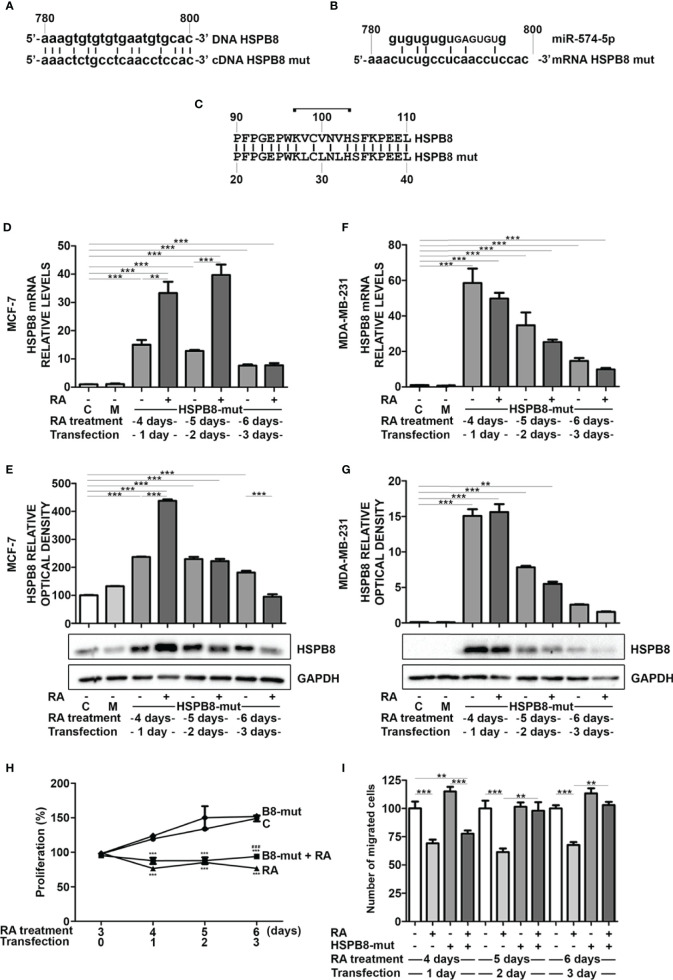
Effects of RA treatment in wfi 0-7 cells overexpressing miR-574-5p mutated HSPB8 variant. **(A)** Alignment of the ORF region (between 780 and 800bp) of wild-type HSPB8 DNA with mutated HSPB8 DNA. **(B)** Alignment of miR-574-5p RNAs with mutated HSPB8 mRNA ORF region (between 780 and 800 bp), capital letters indicate the miRNA *seed sequence*. **(C)** Alignment of wild-type HSPB8 protein sequences with the mutated one, bracket indicates the aminoacids coded by the mRNA region between 780 and 800 bp. **(D)** HSPB8 mRNA measured by RT-qPCR analysis in MCF-7 cells. **(E)** HSPB8 variant protein levels measured by western blot analysis in MCF-7 cells. **(F)** HSPB8 mRNA measured by RT-qPCR analysis in MDA-MB-231 cells. **(G)** HSPB8 variant protein levels measured by western blot analysis in MDA-MB-231 cells. For **(D–G)** untreated cells (C), mock-transfected cells (M) or cells treated up to 6 days with 1μM RA and transfected at day 3 with the plasmid overexpressing miR-574-5p mutated HSPB8 variant. Analyses were performed at 1, 2 and 3 days after transfection. **(H)** Proliferation of MCF-7 cells overexpressing the miR-574-5p mutated HSPB8 variant for 3 days and treated with 1μM RA up to 6 days, values are expressed as percentage on day 3 of RA treatment. **(I)** Migration of MCF-7 cells overexpressing the miR-574-5p mutated HSPB8 variant for 3 days and treated with 1μM RA up to 6 days, values are expressed as number of migrated cells (% *vs* untransfected control). **p < 0.01; ***p < 0.005 in all charts. Graph bars represent the mean of three independent experiments.

To deeply assess whether miR-574-5p mediates the RA effect on HSPB8 levels, we edited miR-574-5p target sequence from HSPB8-coding plasmid to avoid miRNA interaction. We designed the mutation in order to minimize changes in protein structure that might alter its functions and/or turnover. We preferentially removed guanine and cytosine to weaken miRNA/mRNA interaction in order to reduce the highest hydrogen bonds number changing the lowest base pairs (**Figures 4A, B**). As shown in **Figure 4C**, the HSPB8 mutated variant has three Val-to-Leu conservative replacements in positions 98, 100 and 102. Since miR-574-5p target region is localized in the first part of the alpha-crystalline domain, that is a well-structured domain essential for HSPB8 activity, we initially ruled out that the introduced mutations did not affect the expression level/stability and the activity of mutant HSPB8 (**Figure S5**). Then, we overexpressed HSPB8 mutated variant in both MCF-7 and MDA-MB-231 cells, and we analyzed the effect of RA treatment on the overall *HSPB8* mRNA and protein levels. Surprisingly, RA treatment correlated with a further increase of *HSPB8* mRNA levels at 1 and 2 days after transfection (**Figure 4D**), while HSPB8 protein levels were enhanced only at day 1 after transfection in MCF-7 cells (**Figure 4E**). Interestingly, this unexpected stimulatory effect of RA was not observed in MDA-MB-231 cells (**Figures 4F, G**). Since RA treatment in MCF-7 cells overexpressing HSPB8 variant did not cause a reduction of total HSPB8 levels, that remained elevated up to 3 days after transfection, it was possible to assess whether RA treatment was able to reduce MCF-7 cell proliferation even in presence of high HSPB8 protein levels. The data in **Figure 4H** showed that the presence of overexpressed HSPB8 variant did not change the proliferative capacity of MCF-7 cells and that RA maintained its antiproliferative activity even under these conditions. Finally, we also assessed RA antimigratory capacity of MCF-7 cells in the presence of the HSPB8 variant. We found that RA was able to reduce the migratory capacity of MCF-7 cells from 4 to 6 days of treatment. Interestingly, this effect was completely counteracted by HSPB8 variant starting from the fifth day of treatment and after 2 days post transfection (**Figure 4I**).

## Discussion

The study here described is based on our previous demonstration that HSPB8 modulates the proliferation and migration of ER^+^ BC MCF-7 cells. These cells express HSPB8 protein and mRNA at higher levels compared to ER^-^ BC MDA-MB-231 cells (21). Moreover, estrogen-induced HSPB8 expression is absent in MDA-MB-231 cells (21). This suggests that *HSPB8* translation is differentially modulated by transcriptional regulatory mechanisms in the two cell types. It is also known that these cells are differently sensitive to RA treatment, that selectively affects the expression of many genes (31) and miRNAs (11).

Therefore, we decided to analyze the direct effect of RA on endogenous *HSPB8* gene expression in MCF-7 cells and on overexpressed HSPB8 in both MCF-7 and MDA-MB-231 cells. We clearly observed that RA reduces *HSPB8* and *BAG3* gene expression in MCF-7 cells. Since HSPB8 has been shown to induce MCF-7 cell proliferation (21), we postulated that the well-known antiproliferative action exerted by RA on BC cells may also take place through the downregulation of HSPB8 and its co-chaperone BAG3. A possible mechanism of action of RA on MCF-7 proliferation is the mitotic spindle disorganization and incorrect placement of chromosomes in dividing HSPB8-depleted cells. In fact, the HSPB8-BAG3 complex regulates actin dynamics during mitosis by influencing spindle orientation, a process required for chromosome alignment at the metaphase plate and chromosome segregation (30), and already proven to be a target for BC therapy (32). *HSPB8* or *BAG3* silencing resulted in a disorganization of actin-rich retraction fibers and altered spindle orientation, so the HSPB8-BAG3 complex mediates the protein quality control mechanism during mitotic processes activated in proliferating cells (30).

We found that RA inhibits *HSPB8* expression acting on its promoter when its expression is enhanced by 17β-estradiol, known to be a powerful positive regulator of *HSPB8* expression (21, 22); these data agree with other reports demonstrating the antagonism between RAR and ERs on their DNA responsive elements (8). Accordingly to other Authors (23), we also reported that the stimulatory action of 17β-estradiol exerted on MCF-7 cell proliferation is antagonized by RA treatment, and that RA antiproliferative effect is exerted only at a late stage after treatment. We also determined whether RA effects on MCF-7 cells occur even in the presence of HSPB8 overexpression. Surprisingly, high HSPB8 protein levels achieved upon HSPB8 overexpression were reduced by RA treatment. Interestingly, this effect occurred when HSPB8 expression was driven by the exogenous CMV promoter in the encoding plasmid lacking the human *HSPB8* promoter. We excluded that RA acts on the CMV promoter using the same promoter to drive the expression of the β-galactosidase. In addition, MDA-MB-231 cells transfected with the same HSPB8-coding plasmid did not show any decrease of HSPB8 protein levels upon RA treatment. Thus, RA must post-transcriptionally act on *HSPB8* mRNA through a RA-induced factor in MCF-7 cells (and not in MDA-MB-231 cells), such as a miRNA, capable of decreasing *HSPB8* mRNA levels and preventing its translation into the protein. Some miRNAs are capable of binding to *HSPB8* mRNA (33, 34), but those of our interest should be also regulated by RA. MiRNAs targeting the 3’-UTR or 5’-UTR were excluded since both regions are absent in our HSPB8 plasmid; therefore the putative miRNA must directly target the ORF region of *HSPB8* mRNA. *In silico* analysis showed some miRNAs interacting with the ORF region of *HSPB8* mRNA and among these, our attention focused on miR-297 and 574-5p, both able to bind in the same trait between 780bp and 800bp. So far, miR-297 has never been reported in any BC study, even if it was identified as tumor suppressor in prostate cancer (35), in colorectal cancer (36) and glioblastoma (37), while in pulmonary adenocarcinoma it has an oncogenic effect (38). Conversely, miR-574-5p is highly expressed in the triple negative BC SKBR3 cell line (11) where it acts as tumor suppressor (39, 40). We found that miR-297 and miR-574-5p are expressed in MCF-7 and MDA-MB-231 cells, but only miR-574-5p is downregulated by RA specifically in MCF-7 cells in which it correlates with the reduction of HSPB8 expression. The opposite has been observed in MDA-MB-231 cells, that express higher levels of miR-574-5p possibly compensating for the lower levels of *HSPB8* mRNA (21). Thanks to a *HSPB8* cDNA mutated in the putative target sequence of miR-574-5p, we confirmed that this *HSPB8* ORF specific region is involved in RA-mediated downregulation of *HSPB8*, since RA treatment is not able to affect mutated *HSPB8* mRNA and protein levels in MCF-7 cells. We also confirmed that RA reduces BC cells migration (41) and counteracts the pro-migratory activity of HSPB8 in MCF-7 cells (21). We characterized the possible physiological antagonism between miR-574-5p and the HSPB8 mutated variant on RA antimigratory effect, further proving that high HSPB8 levels are counteracted by RA, making cells refractory to its action on migration.

**Figure 5:**
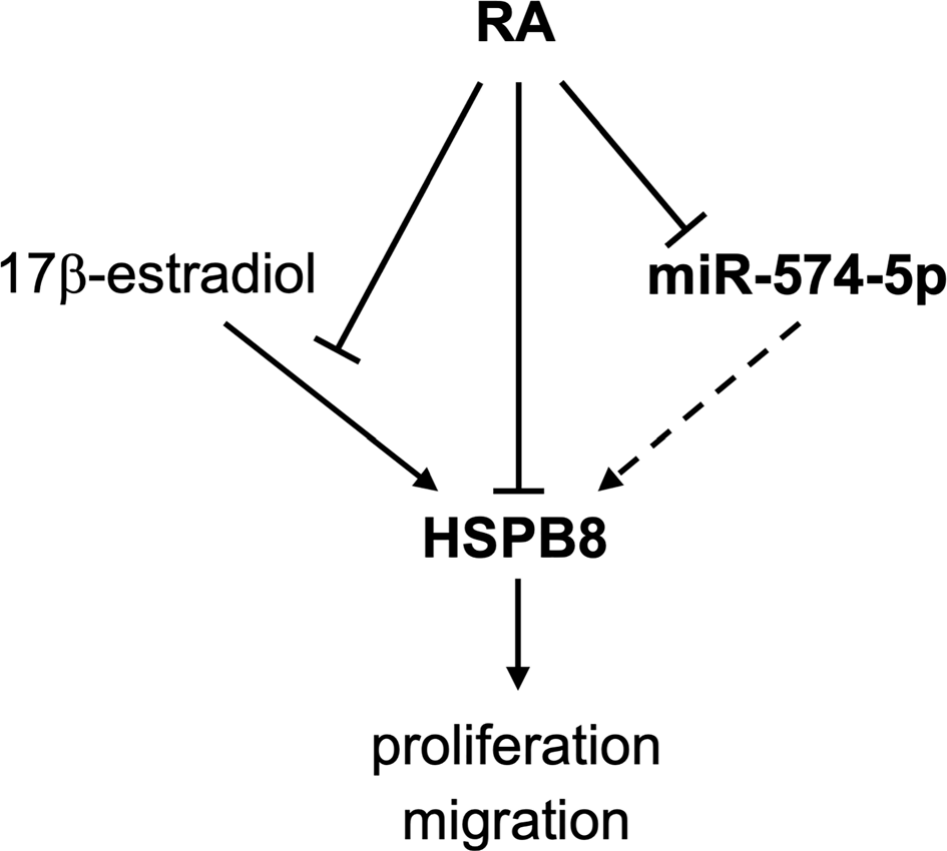
Schematic representation of the RA effects on MCF-7 cells unveiled in this study.

The data here reported (graphically summarized in **Figure 5**) suggest that in MCF-7 cells RA reduces *HSPB8* gene expression modulating the proliferative and migratory activity of this cell line. RA inhibitory action on MCF-7 proliferation and migration is also exerted in the presence of high levels of HSPB8. We identify the miR-574-5p as a modulator of HSPB8 expression by its binding to *HSPB8* ORF.

## Data Availabilty Statement

The original contributions presented in the study are included in the article/**Supplementary Material**. Further inquiries can be directed to the corresponding author.

## Author Contributions

Conceptualization, MP, RC, AP, and EM. Methodology, MG, VC, and PR. Validation BT, EC, and VF. Investigation, MCh and MCo. Writing—Original Draft Preparation, EM, MP, RC, and AP. Writing—Review & Editing, VC, PR, BT, VF, MCh, MCo, and MG. Supervision, AP. All authors contributed to the article and approved the submitted version.

## Funding

This research was funded by: Fondazione Telethon, Italy (n. GGP14039 to AP, GGP19128 to AP); Kennedy’s disease association (2018 grant to RC); Fondazione Cariplo, Italy (n. 2014-0686 to AP; n. 2017-0747 to VC); Fondazione AriSLA, Italy (n. ALS_HSPB8 to AP; ALS_Granulopathy to AP; MLOpathy to AP; Target-RAN to AP); Association Française contre les Myopathies, France (AFM Telethon n. 16406 to AP); Università degli Studi di Milano e piano di sviluppo UNIMI—linea B (to VC and PR); Bando Straordinario per Progetti Interdipartimentali (Bando SEED 2019: #TDP-43-iPSC to VC) Italian Ministry of University and Research (MIUR), PRIN—Progetti di ricerca di interesse nazionale (n. 2015LFPNMN to AP; n. 2017F2A2C5 to AP); Fondo per il Finanziamento delle Attività Base di Ricerca (FFABR) (MIUR, to MG, EM, and to PR); Agenzia Italiana del Farmaco (AIFA) (Co_ALS to AP); Fondazione Regionale per la Ricerca Biomedica (FRRB) (Regione Lombardia, TRANS_ALS, project nr. 2015-0023, to AP). EU Joint Programme—Neurodegenerative Disease Research (JPND) project. The project is supported through the following funding organizations under the aegis of JPND—www.jpnd.eu. This project has received funding from the European Union’s Horizon 2020 research and innovation programme under grant agreement N° 643417 [Grant ID: 01ED1601A, CureALS] (to AP); Italian Ministry of University and Research [Progetto Dipartimenti di Eccellenza].

## Conflict of Interest

The authors declare that the research was conducted in the absence of any commercial or financial relationships that could be construed as a potential conflict of interest.

## References

[1] Ferlay J Steliarova-Foucher E Lortet-Tieulent J Rosso S Coebergh JW Comber H. Cancer Incidence and Mortality Patterns in Europe: Estimates for 40 Countries in 2012. Eur J Cancer (2013) 49:1374–403. 10.1016/j.ejca.2012.12.027 23485231

[2] Majumder A Singh M Tyagi SC. Post-Menopausal Breast Cancer: From Estrogen to Androgen Receptor. Oncotarget (2017) 8:102739–58. 10.18632/oncotarget.22156 PMC5731994 29254284

[3] Radhi S. Molecular Changes During Breast Cancer and Mechanisms of Endocrine Therapy Resistance. Prog Mol Biol Transl Sci (2016) 144:539–62. 10.1016/bs.pmbts.2016.09.009 27865467

[4] Ma XJ Wang Z Ryan PD Isakoff SJ Barmettler A Fuller A. A Two-Gene Expression Ratio Predicts Clinical Outcome in Breast Cancer Patients Treated With Tamoxifen. Cancer Cell (2004) 5:607–16. 10.1016/j.ccr.2004.05.015 15193263

[5] Tang XH Gudas LJ. Retinoids, Retinoic Acid Receptors, and Cancer. Annu Rev Pathol (2011) 6:345–64. 10.1146/annurev-pathol-011110-130303 21073338

[6] Costantini L Molinari R Farinon B Merendino N. Retinoic Acids in the Treatment of Most Lethal Solid Cancers. J Clin Med (2020) 9:360. 10.3390/jcm9020360 PMC7073976 32012980

[7] Garattini E Bolis M Garattini SK Fratelli M Centritto F Paroni G. Retinoids and Breast Cancer: from Basic Studies to the Clinic and Back Again. Cancer Treat Rev (2014) 40:739–49. 10.1016/j.ctrv.2014.01.001 24480385

[8] Hua S Kittler R White KP. Genomic Antagonism Between Retinoic Acid and Estrogen Signaling in Breast Cancer. Cell (2009) 137:1259–71. 10.1016/j.cell.2009.04.043 PMC3374131 19563758

[9] Saumet A Vetter G Bouttier M Antoine E Roubert C Orsetti B. Estrogen and Retinoic Acid Antagonistically Regulate Several microRNA Genes to Control Aerobic Glycolysis in Breast Cancer Cells. Mol Biosyst (2012) 8:3242–53. 10.1039/c2mb25298h 23064179

[10] Terao M Fratelli M Kurosaki M Zanetti A Guarnaccia V Paroni G. Induction of miR-21 by Retinoic Acid in Estrogen Receptor-Positive Breast Carcinoma Cells: Biological Correlates and Molecular Targets. J Biol Chem (2011) 286:4027–42. 10.1074/jbc.M110.184994 PMC3030403 21131358

[11] Fisher JN Terao M Fratelli M Kurosaki M Paroni G Zanetti A. MicroRNA Networks Regulated by All-Trans Retinoic Acid and Lapatinib Control the Growth, Survival and Motility of Breast Cancer Cells. Oncotarget (2015) 6:13176–200. 10.18632/oncotarget.3759 PMC4537007 25961594

[12] Ueda S Takanashi M Sudo K Kanekura K Kuroda M. miR-27a Ameliorates Chemoresistance of Breast Cancer Cells by Disruption of Reactive Oxygen Species Homeostasis and Impairment of Autophagy. Lab Invest (2020) 100:863–73. 10.1038/s41374-020-0409-4 32066826

[13] Maycotte P Thorburn A. Targeting Autophagy in Breast Cancer. World J Clin Oncol (2014) 5:224–40. 10.5306/wjco.v5.i3.224 PMC4127596 25114840

[14] Cook KL Shajahan AN Clarke R. Autophagy and Endocrine Resistance in Breast Cancer. Expert Rev Anticancer Ther (2011) 11:1283–94. 10.1586/era.11.111 PMC3701722 21916582

[15] Arndt V Dick N Tawo R Dreiseidler M Wenzel D Hesse M. Chaperone-Assisted Selective Autophagy is Essential for Muscle Maintenance. Curr Biol (2010) 20:143–8. 10.1016/j.cub.2009.11.022 20060297

[16] Cristofani R Piccolella M Crippa V Tedesco B Montagnani Marelli M Poletti A. The Role of HSPB8, a Component of the Chaperone-Assisted Selective Autophagy Machinery, in Cancer. Cells (2021) 10:335. 10.3390/cells10020335 PMC7915307 33562660

[17] Crippa V Carra S Rusmini P Sau D Bolzoni E Bendotti C. A Role of Small Heat Shock Protein B8 (HspB8) in the Autophagic Removal of Misfolded Proteins Responsible for Neurodegenerative Diseases. Autophagy (2010) 6:958–60. 10.4161/auto.6.7.13042 20699640

[18] Crippa V Sau D Rusmini P Boncoraglio A Onesto E Bolzoni E. The Small Heat Shock Protein B8 (HspB8) Promotes Autophagic Removal of Misfolded Proteins Involved in Amyotrophic Lateral Sclerosis (ALS). Hum Mol Genet (2010) 19:3440–56. 10.1093/hmg/ddq257 20570967

[19] Cristofani R Crippa V Rusmini P Cicardi ME Meroni M Licata NV. Inhibition of Retrograde Transport Modulates Misfolded Protein Accumulation and Clearance in Motoneuron Diseases. Autophagy (2017) 13:1280–303. 10.1080/15548627.2017.1308985 PMC5584856 28402699

[20] Cristofani R Rusmini P Galbiati M Cicardi ME Ferrari V Tedesco B. The Regulation of the Small Heat Shock Protein B8 in Misfolding Protein Diseases Causing Motoneuronal and Muscle Cell Death. Front Neurosci (2019) 13:796. 10.3389/fnins.2019.00796 31427919 PMC6688727

[21] Piccolella M Crippa V Cristofani R Rusmini P Galbiati M Cicardi ME. The Small Heat Shock Protein B8 (HSPB8) Modulates Proliferation and Migration of Breast Cancer Cells. Oncotarget (2017) 8:10400–15. 10.18632/oncotarget.14422 PMC5354667 28060751

[22] Sun X Fontaine JM Bartl I Behnam B Welsh MJ Benndorf R. Induction of Hsp22 (HspB8) by Estrogen and the Metalloestrogen Cadmium in Estrogen Receptor-Positive Breast Cancer Cells. Cell Stress Chaperones (2007) 12:307–19. 10.1379/CSC-276.1 PMC2134793 18229450

[23] Salvatori L Ravenna L Caporuscio F Principessa L Coroniti G Frati L. Action of Retinoic Acid Receptor on EGFR Gene Transactivation and Breast Cancer Cell Proliferation: Interplay With the Estrogen Receptor. BioMed Pharmacother (2011) 65:307–12. 10.1016/j.biopha.2011.03.007 21705183

[24] Cashman NR Durham HD Blusztajn JK Oda K Tabira T Shaw IT. Neuroblastoma X Spinal Cord (NSC) Hybrid Cell Lines Resemble Developing Motor Neurons. Dev Dyn (1992) 194:209–21. 10.1002/aja.1001940306 1467557

[25] Cristofani R Crippa V Vezzoli G Rusmini P Galbiati M Cicardi ME. The Small Heat Shock Protein B8 (HSPB8) Efficiently Removes Aggregating Species of Dipeptides Produced in C9ORF72-related Neurodegenerative Diseases. Cell Stress Chaperones (2018) 23:1–12. 10.1007/s12192-017-0806-9 28608264 PMC5741577

[26] Carra S Sivilotti M Chavez Zobel AT Lambert H Landry J. HspB8, a Small Heat Shock Protein Mutated in Human Neuromuscular Disorders, has In Vivo Chaperone Activity in Cultured Cells. Hum Mol Genet (2005) 14:1659–69. 10.1093/hmg/ddi174 15879436

[27] Balatti V Oghumu S Bottoni A Maharry K Cascione L Fadda P. Microrna Profiling of Salivary Duct Carcinoma Versus Her2/Neu Overexpressing Breast Carcinoma Identify miR-10a as a Putative Breast Related Oncogene. Head Neck Pathol (2018) 13:344–54. 10.1007/s12105-018-0971-x PMC6684709 30259272

[28] Chang JT Wang F Chapin W Huang RS. Identification of MicroRNAs as Breast Cancer Prognosis Markers Through the Cancer Genome Atlas. PloS One (2016) 11:e0168284. 10.1371/journal.pone.0168284 27959953 PMC5154569

[29] Rusmini P Bolzoni E Crippa V Onesto E Sau D Galbiati M. Proteasomal and Autophagic Degradative Activities in Spinal and Bulbar Muscular Atrophy. Neurobiol Dis (2010) 40:361–9. 10.1016/j.nbd.2010.06.016 20621188

[30] Fuchs M Luthold C Guilbert SM Varlet AA Lambert H Jette A. A Role for the Chaperone Complex BAG3-HSPB8 in Actin Dynamics, Spindle Orientation and Proper Chromosome Segregation During Mitosis. PloS Genet (2015) 11:e1005582. 10.1371/journal.pgen.1005582 26496431 PMC4619738

[31] Bolis M Garattini E Paroni G Zanetti A Kurosaki M Castrignano T. Network-Guided Modeling Allows Tumor-Type Independent Prediction of Sensitivity to All-Trans-Retinoic Acid. Ann Oncol (2017) 28:611–21. 10.1093/annonc/mdw660 PMC5834014 27993792

[32] Gulluni F Martini M De Santis MC Campa CC Ghigo A Margaria JP. Mitotic Spindle Assembly and Genomic Stability in Breast Cancer Require PI3K-C2alpha Scaffolding Function. Cancer Cell (2017) 32:444–59 e7. 10.1016/j.ccell.2017.09.002 29017056

[33] Kong F He S Shen X Li L Fang J Lian M. Integrated Analysis of Different mRNA and miRNA Profiles in Human Hypopharyngeal Squamous Cell Carcinoma Sensitive and Resistant to Chemotherapy. Neoplasma (2020) 67:473–83. 10.4149/neo_2020_190320N249 32064881

[34] Yuan J Wu Y Li L Liu C. MicroRNA-425-5p Promotes Tau Phosphorylation and Cell Apoptosis in Alzheimer’s Disease by Targeting Heat Shock Protein B8. J Neural Transm (Vienna) (2020) 127:339–46. 10.1007/s00702-019-02134-5 31919655

[35] Fang Z Xu C Li Y Cai X Ren S Liu H. A Feed-Forward Regulatory Loop Between Androgen Receptor and PlncRNA-1 Promotes Prostate Cancer Progression. Cancer Lett (2016) 374:62–74. 10.1016/j.canlet.2016.01.033 26808578

[36] Xu K Liang X Shen K Cui D Zheng Y Xu J. miR-297 Modulates Multidrug Resistance in Human Colorectal Carcinoma by Down-Regulating MRP-2. Biochem J (2012) 446:291–300. 10.1042/BJ20120386 22676135

[37] Kefas B Floyd DH Comeau L Frisbee A Dominguez C Dipierro CG. A miR-297/hypoxia/DGK-alpha Axis Regulating Glioblastoma Survival. Neuro Oncol (2013) 15:1652–63. 10.1093/neuonc/not118 PMC3829588 24158111

[38] Sun Y Zhao J Yin X Yuan X Guo J Bi J. miR-297 Acts as an Oncogene by Targeting GPC5 in Lung Adenocarcinoma. Cell Prolif (2016) 49:636–43. 10.1111/cpr.12288 PMC6496124 27554041

[39] Zhang KJ Hu Y Luo N Li X Chen FY Yuan JQ. miR5745p Attenuates Proliferation, Migration and EMT in Triplenegative Breast Cancer Cells by Targeting BCL11A and SOX2 to Inhibit the SKIL/TAZ/CTGF Axis. Int J Oncol (2020) 56:1240–51. 10.3892/ijo.2020.4995 32319565

[40] Wang PS Chou CH Lin CH Yao YC Cheng HC Li HY. A Novel Long Non-Coding RNA linc-ZNF469-3 Promotes Lung Metastasis Through miR-574-5p-ZEB1 Axis in Triple Negative Breast Cancer. Oncogene (2018) 37:4662–78. 10.1038/s41388-018-0293-1 29755127

[41] Flamini MI Gauna GV Sottile ML Nadin BS Sanchez AM Vargas-Roig LM. Retinoic Acid Reduces Migration of Human Breast Cancer Cells: Role of Retinoic Acid Receptor Beta. J Cell Mol Med (2014) 18:1113–23. 10.1111/jcmm.12256 PMC4508151 24720764

