# Supplementary material for: Retinoic Acid Downregulates HSPB8 Gene Expression in Human Breast Cancer Cells MCF-7
Source: Front Oncol. 2021 May 31;11:652085. doi: 10.3389/fonc.2021.652085 (PMC8201400; doi:10.3389/fonc.2021.652085)
Supplement: Supplementary file 1 [file DataSheet_1.docx]

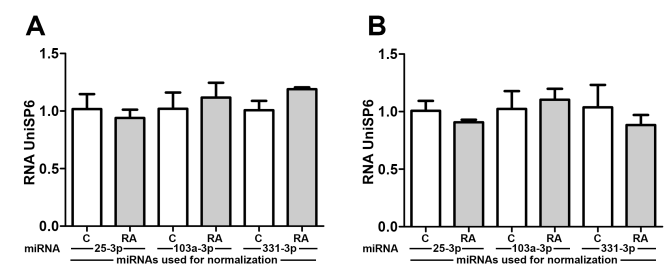


**Figure S1: Recovery of UniSP6 RNA Spike-in-template from cell lysate and miRNAs analysis**. **A,** Equal amounts of UniSP6 RNA Spike-in-template was added in MCF-7 cells lysates and measured by RT-qPCR analysis utilizing three miRNA as housekeeping: miR-25-3p, miR-103a-3p and miR-331-3p. **B,** Equal amounts of UniSP6 RNA Spike-in-template was added in MDA-MB-231 cells lysates and measured by RT-qPCR analysis utilizing three microRNA as housekeeping: miR-25-3p, miR-103a-3p and miR-331-3p.

To validate the realtime system used for miRNAs analysis we measured the levels of UniSp6RNA, a control RNA provided with the miRCURY LNA miRNA PCR Starter Kit (Qiagen, Ref 339320) added before the reverse transcription in equal amounts to all samples obtained from MCF-7 and MDA-MB-231 cells treated or not for 4 days with RA. Since it is known that in miRNAs RT-qPCR analysis there is not a fully reliable and suitable reference miRNA, we normalized UniSP6RNA levels using three different housekeepings miRNAs: miR-25-5p, miR-103a-3p and miR-331-3p.

The results showed that UniSp6RNA levels measured in samples from both MCF-7 and MDA-MB-231 untreated cells are identical to those found in samples derived from RA-treated cells (**Fig**. **2H** and **I**). Since an identical amount of UniSP6RNA was added to all samples, this result clearly indicates that all reference miRNAs used in the analysis did not change as a result of RA treatment and that they can be utilized to normalize the levels of the two miRNAs being used in this experiment.

**
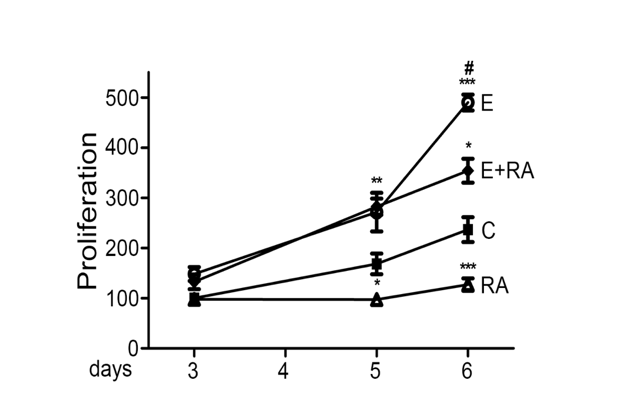
**

**Figure S2: RA modulation of MCF-7 proliferation**. Cell proliferation of MCF-7 treated with 1μM RA and 10nM 17β-estradiol (E) up to 6 days measured by MTT assay, values are expressed as percentage on third day of untreated (C) cells. *p<0.05, **p<0.01 and ***p<0.005 in all charts. *p<0.05; **p<0.01; ***p<0.005 *vs* control and #p<0.05 *vs* E+RA.


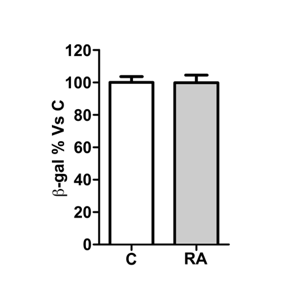


**Figure S3: RA modulation of CMV promoter**. MCF-7 cells transfected with pCMV-β-gal and treated with 1μM RA. Graph bars represent the mean of three independent experiments.


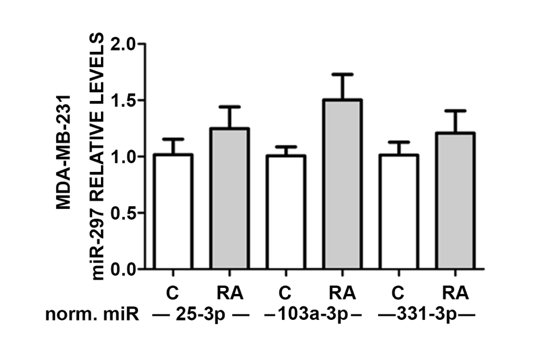


**Figure S4: miR-297 expression in MDA-MB-231 cells**. RT-qPCR analysis of miR-297 in MDA-MB-231 cells.

**
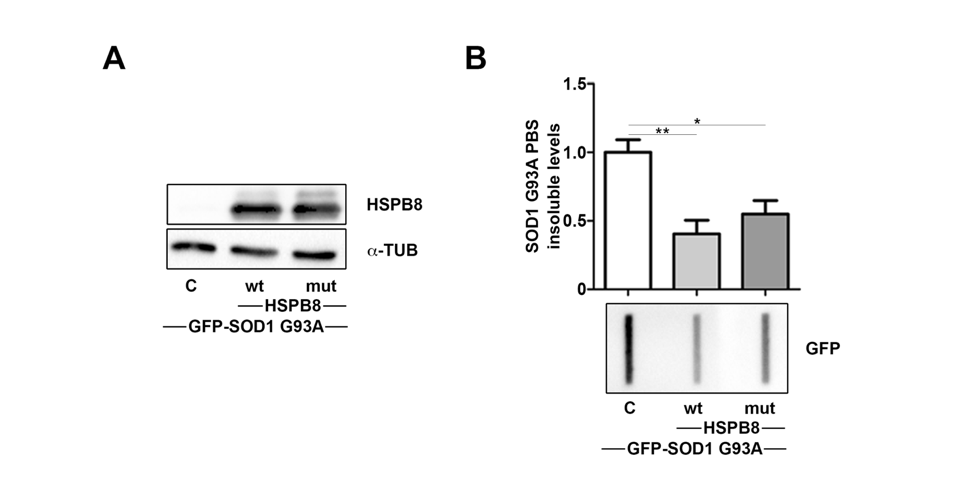
­**

**Figure S5: Evaluation of HSPB8 mutated variant activity**. **A**, Western blot assay of NSC34 cells overexpressing the miR-574-5p mutated HSPB8 variant and the G93A-SOD1. **B**, Filter retardation assay of NSC34 cells overexpressing the miR-574-5p mutated HSPB8 varian­­t and the G93A-SOD1.

We evaluated HSPB8 capability to promote the autophagic removal of misfolded proteins. In fact, HSPB8 overexpression is able to reduce the PBS-insoluble fraction of the G93A-SOD1 protein responsible for amyotrophic lateral sclerosis (ALS) (17). The overexpression of HSPB8 mutated variant in motor neuron cells (NSC34) transfected with G93A-SOD1 showed the same protein levels to those observed in wt overexpressed samples (**Fig. S5 A**). Moreover, the HSPB8 mutated variant retained the capability to reduce the PBS-insoluble species of the G93A-SOD1 (**Fig. S5 B**) observed in wt HSPB8 samples.
